# PSMA, EpCAM, VEGF and GRPR as Imaging Targets in Locally Recurrent Prostate Cancer after Radiotherapy

**DOI:** 10.3390/ijms15046046

**Published:** 2014-04-10

**Authors:** Maxim Rybalov, Hildo J. K. Ananias, Hilde D. Hoving, Henk G. van der Poel, Stefano Rosati, Igle J. de Jong

**Affiliations:** 1 Department of Urology, University Medical Center Groningen, University of Groningen, P.O. Box 30.001, Groningen 9700 RB NL, The Netherlands; E-Mails: maxrybalov@mail.ru (M.R.); h.j.k.ananias@umcg.nl (H.J.K.A.); h.d.hoving@umcg.nl (H.D.H.); 2 Department of Urology, the Netherlands Cancer Institute, Antoni van Leeuwenhoek Hospital, Plesmanlaan 121, Amsterdam 1066 CX, The Netherlands; E-Mail: h.vd.poel@nki.nl; 3 Department of Pathology, University Medical Center Groningen, University of Groningen, P.O. Box 30.001, Groningen 9700 RB NL, The Netherlands; E-Mail: s.rosati@umcg.nl

**Keywords:** prostate cancer, immunohistochemistry, PSMA, EpCAM, VEGF, GRPR

## Abstract

In this retrospective pilot study, the expression of the prostate-specific membrane antigen (PSMA), the epithelial cell adhesion molecule (EpCAM), the vascular endothelial growth factor (VEGF) and the gastrin-releasing peptide receptor (GRPR) in locally recurrent prostate cancer after brachytherapy or external beam radiotherapy (EBRT) was investigated, and their adequacy for targeted imaging was analyzed. Prostate cancer specimens were collected of 17 patients who underwent salvage prostatectomy because of locally recurrent prostate cancer after brachytherapy or EBRT. Immunohistochemistry was performed. A pathologist scored the immunoreactivity in prostate cancer and stroma. Staining for PSMA was seen in 100% (17/17), EpCAM in 82.3% (14/17), VEGF in 82.3% (14/17) and GRPR in 100% (17/17) of prostate cancer specimens. Staining for PSMA, EpCAM and VEGF was seen in 0% (0/17) and for GRPR in 100% (17/17) of the specimens’ stromal compartments. In 11.8% (2/17) of cases, the GRPR staining intensity of prostate cancer was higher than stroma, while in 88.2% (15/17), the staining was equal. Based on the absence of stromal staining, PSMA, EpCAM and VEGF show high tumor distinctiveness. Therefore, PSMA, EpCAM and VEGF can be used as targets for the bioimaging of recurrent prostate cancer after EBRT to exclude metastatic disease and/or to plan local salvage therapy.

## 1. Introduction

Prostate cancer is the most commonly diagnosed cancer among men, and its incidence rates remain as the highest in many regions of the world [1]. About 18% of patients with localized disease develop a prostate-specific antigen (PSA) recurrence within five years after brachytherapy and external beam radiotherapy (EBRT) with doses higher than 72 Gy. This is in contrast to 49% of patients who were treated with doses less than 72 Gy [2]. At present, there is no imaging modality that can accurately discriminate between locally and distant recurrent prostate cancer after treatment with curative intent. Selecting patients for salvage cryotherapy of the prostate or salvage prostatectomy by excluding distant metastases proves a diagnostic challenge.

New diagnostic techniques are needed to improve the imaging of recurrent prostate cancer. Furthermore, although early-stage and locally recurrent prostate cancer can be cured, treatment of metastasized disease is currently only palliative, making it important for new therapeutic applications to be devised [3].

Significant expression of certain antigens in prostate cancer as compared to normal tissue could be used for antigen-targeted imaging or therapy. Numerous pre-clinical and clinical trials focused on this topic have already shown promising results [4–8].

Among the antigens used for diagnostic applications, the prostate-specific membrane antigen (PSMA) was demonstrated to be a useful target [9–13]. PSMA is a Type II integral membrane glycoprotein, which is overexpressed in prostate cancer in comparison to benign prostate tissue [11,14,15]. Next to expression in prostate, PSMA was found to be expressed in the neovasculature of solid tumors in comparison to normal vasculature [16,17]. The biological role of PSMA is not completely understood [10,18,19].

Ross *et al.* demonstrated a significant correlation between PSMA expression in prostate cancer and the Gleason score, pathological stage and biochemical recurrence [11]. Indium-111 capromab pendetide (ProstaScint^®^) is a radiolabelled antibody directed against PSMA. Correlation of scan results with pathological specimens suggests that ProstaScint is able to detect soft tissue metastases [20–23]. However, for routine use in clinical practice, the sensitivity of ProstaScint is not high enough, because the antibody targets the intracellular epitope of PSMA, thereby probably targeting only damaged or necrotic/apoptotic cells. Furthermore, the role of ProstaScint in the diagnosis of recurrent disease has to be elucidated [24].

Another antigen that can be used as an imaging target is the epithelial cell adhesion molecule (EpCAM). EpCAM is a transmembrane glycoprotein, which is highly expressed in rapidly proliferating tumors of epithelial origin [25–27]. This protein is found to be strongly expressed in several carcinomas [28–31]. In normal epithelium, there is a lower expression of EpCAM [32]. EpCAM mediates epithelial-specific intercellular cell-adhesion. Next, it is suggested that EpCAM is involved in cell migration, signaling, proliferation and differentiation [33]. The expression of EpCAM is inversely related to prognosis in several carcinomas [33]. For prostate cancer, this relation is controversial [32,34].

Signal protein vascular endothelial growth factor (VEGF) and its receptors are involved in (tumor-related) angiogenesis [35,36]. VEGF is overexpressed in a variety of tumors, including gliomas, breast, renal cell and hepatocellular cancer [37]. VEGF is a potential target, as its expression has also been demonstrated in prostate cancer [38,39]. The expression of VEGF in normal prostate, benign prostate hyperplasia and prostate cancer in relation to tumor grade is inconsistent in the current literature [7,40–49]. As for EpCAM, the prognostic value of VEGF expression is controversial [50–53].

The gastrin-releasing peptide receptor (GRPR) can be a promising imaging target. GRPR is a glycosylated seven-transmembrane G-protein coupled receptor, which is expressed in numerous cancers, such as those of the lung, colon and prostate [54–59]. GRPR seems to be overexpressed in prostate cancer in comparison to sparse expression in normal prostate tissue [60–62]. Binding of GRPR stimulates the growth of prostate cancer cells *in vitro* and *in vivo* [63,64]. A significant inverse correlation was found between GRPR expression and an increasing Gleason score [60].

Currently, there is no knowledge about the expression of PSMA, EpCAM, VEGF and GRPR in locally recurrent prostate cancer after brachytherapy or external beam radiotherapy. Therefore, the aim of this pilot study was to investigate the expression of these antigens using immunohistochemistry and to analyze their potency for new diagnostic applications in locally recurrent prostate cancer.

## 2. Results and Discussion

### 2.1. Results

In Table 1, the results of the immunohistochemical staining of different antibodies in prostate cancer specimens are presented.

Overall, staining for PSMA was seen in 100% (17/17), EpCAM in 82.3% (14/17), VEGF in 82.3% (14/17) and GRPR in 100% (17/17) of prostate cancer specimens. Staining for PSMA, EpCAM and VEGF was seen in 0% (0/17) and for GRPR in 100% (17/17) of the specimens’ stromal compartments. Immunohistochemical staining intensity frequency, number and percent are shown in Table 2.

In 11.8% (2/17) of cases, the GRPR staining intensity of prostate cancer was higher than that of stroma. In 88.2% (15/17) of cases, the GRPR staining intensity of prostate cancer was equal to the staining intensity of stroma. Tumor distinctiveness is shown in Table 3.

### 2.2. Staining Pattern

**Figure 1.**
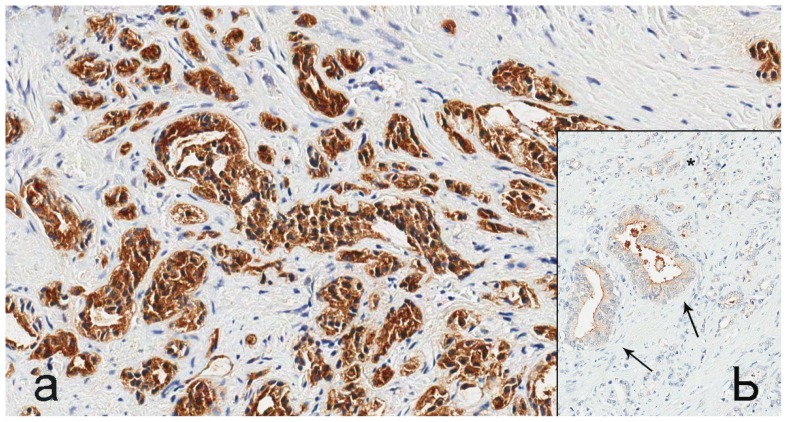
PSMA staining in prostate cancer tissue. (**a**) Membranous and cytoplasmic brown staining for PSMA in prostate cancer cells, 400× magnification; (**b**) focal brown staining for PSMA in prostate cancer cells (arrows) amidst negative cancer tissue (*), 200× magnification.

**Figure 2.**
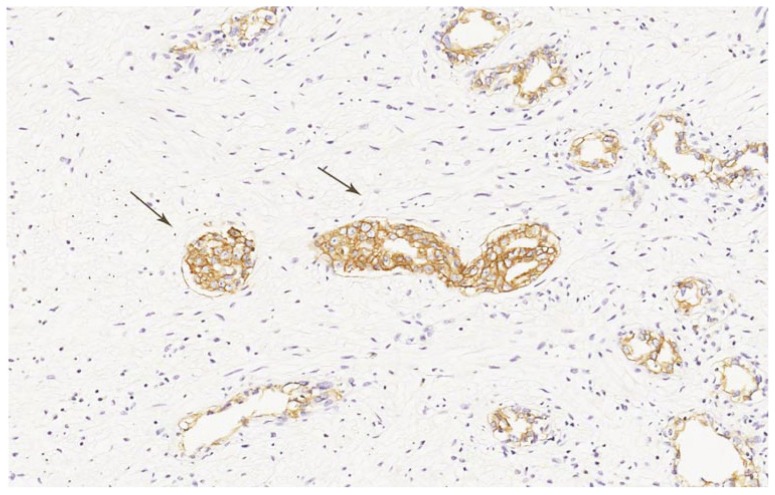
EpCAM staining in prostate cancer tissue. Strong brown cytoplasmic staining for EpCAM in prostate cancer cells (arrows), 400× magnification.

**Figure 3.**
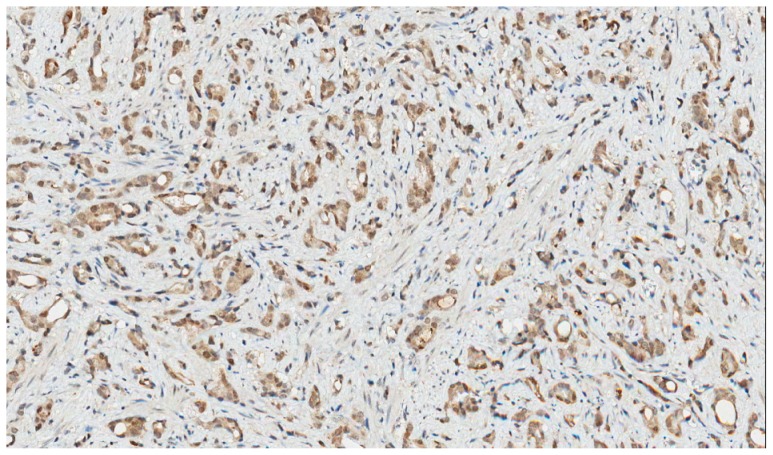
VEGF staining in prostate cancer tissue. Strong brown cytoplasmic staining for VEGF in prostate cancer cells, 200× magnification.

**Figure 4.**
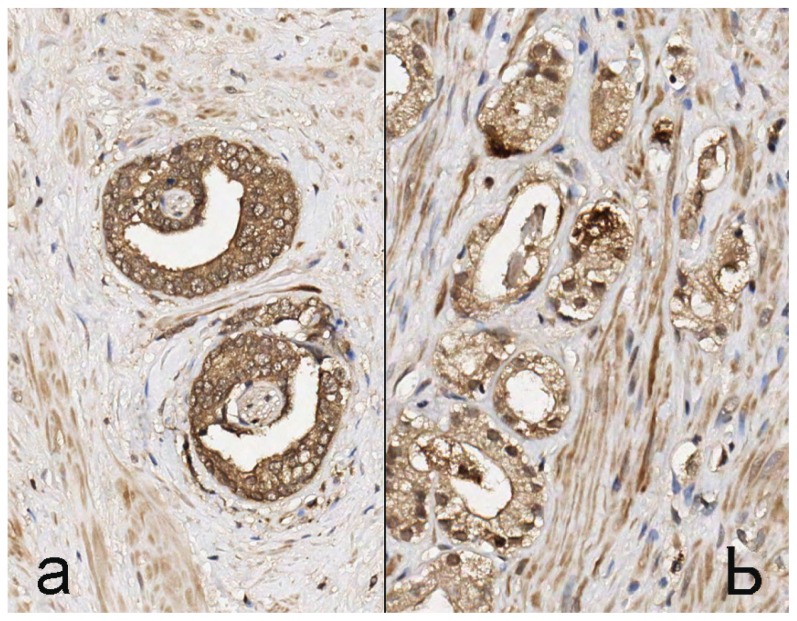
GRPR staining in prostate cancer tissue. (**a**) Strong brown cytoplasmic staining for GRPR in prostate cancer cells and weak background staining of prostate stromal cells; (**b**) equal staining intensity of prostate cancer and stroma, 200× magnification.

PSMA staining in prostate cancer specimens was membranous and cytoplasmic (Figure 1). Six cases with focal uptake amidst negative cancer tissue were observed (Patient No. 2–4, 6, 8, 13) (Figure 1). For PSMA, there was minimal staining of prostatic intraepithelial neoplasia (PIN) and normal prostate epithelium. EpCAM stained cytoplasm and at the basal membrane (Figure 2). VEGF and GRPR staining in prostate cancer tissue was cytoplasmic and diffuse (Figures 3 and 4). Staining of striated muscle was observed for GRPR.

### 2.3. Discussion

This study has shown that PSMA, EpCAM, VEGF and GRPR are expressed in locally recurrent prostate cancer after brachytherapy and external beam radiotherapy. Staining for PSMA and GRPR was observed in all prostate cancer specimens (17/17), while EpCAM and VEGF staining was observed in 82.3% (14/17) of cases (Tables 1 and 2). Staining for PSMA, EpCAM and VEGF was absent (0/17) in specimens’ stromal compartments, while GRPR staining in stroma was observed in 100% (17/17) of cases. In 88.2% (15/17) of cases, the GRPR staining intensity of prostate cancer was equal to the staining intensity of stroma. Staining of striated muscle was observed for GRPR, which is in agreement with the findings of other groups [60–62].

The high expression of PSMA, EpCAM, VEGF and GRPR in locally recurrent prostate cancer after ionizing therapy could have several reasons, which will be postulated next. First, high expression of either antigen is correlated with a tendency for recurrence. This holds true for PSMA, as its expression in primary prostate cancer is positively correlated with factors, like a high Gleason grade, a higher tumor stage and, most important in this case, biochemical recurrence [11,18]. The same goes for EpCAM, as a recent study by Benko *et al.* showed that higher EpCAM expression correlated with a higher Gleason score and a shorter disease-free survival [34]. However, other research groups found conflicting results [26,30,65]. Expression of VEGF in prostate cancer positively correlated with high a Gleason score, lymph node metastases and the progression of disease [66]. Although there is no information about GRPR expression and recurrence, the process might be comparable.

Second is the upregulation of antigens due to radiotherapy. It has been demonstrated that VEGF is upregulated after radiotherapy for rectal cancer [67]. For EpCAM, PSMA and GRPR, this is currently unknown. Third, recurrent cancer could have the tendency for upregulation of certain antigens. In ovarian cancer, EpCAM upregulation was seen in recurrent cancer when compared to primary cancer in matched samples [68], while VEGF levels were significantly higher in recurrent acute lymphoblastic leukemia compared with newly diagnosed cases [69]. For all the postulations, we have no direct evidence, as we did not have prostate cancer biopsy samples before and after radiation therapy that could have been compared to the salvage prostatectomy samples.

The absence of non-specific background staining of normal tissue or prostate stroma for the anti-PSMA, EpCAM and VEGF antibodies proves the specificity for cancer tissue. Background staining for GRPR was evident. Although different protocols were used and different blocking agents were tested, staining of non-cancer tissue remained. Staining of normal prostate and muscle with the anti-GRPR antibody has been described in the literature by other authors [60–62], and at first glance, this might pose a problem for the future use of GRPR as a target for new therapeutic or diagnostic modalities. However, pre-clinical and clinical studies have shown very low uptake of GRPR-targeted bombesin-like radiopharmaceuticals in muscle with high tumor-to-muscle ratios [70].

The current study has several limitations. First, we did not have matched samples of prostate cancer tissue. In an ideal situation, we would have compared prostate biopsy samples at initial diagnosis before radiation therapy, the biopsy samples in which local recurrence was confirmed after radiation therapy and the salvage prostatectomy samples; Second, staining intensity was assessed qualitatively on a scale from zero to three. Although strong staining suggests a higher antigen density than weak staining does, it is impossible to make a comparison between the different antibodies used in this study, as different protocols were used and as there is most likely different antigen sensitivity for each antibody. A quantification of antigen-densities has to be performed to be able to make a comparison between antigens; Third, due to the low number of patients (*n* = 17), the power of the statistics would be too weak, and therefore, we were unable to correlate staining intensity with clinicopathological parameters.

Finally, we did not have the follow-up data to compare antigen expression with outcome.

There is not much knowledge about the expression of antigens in recurrent prostate cancer. Locally recurrent prostate cancer is, to our knowledge, an unexplored field. Therefore, this is the first study to show the data about the expression of PSMA, VEGF, EpCAM and GRPR in locally recurrent prostate cancer after ionizing therapy.

Antigen-based targeted imaging of the prostate could be useful in several scenarios: if salvage local therapy was planned without a biopsy or with repeated negative biopsies, but with a strong suspicion of local recurrence. Other applications for these imaging strategies would be to rule out micrometastatic disease prior to local salvage therapy or the application of salvage focal therapy only to areas of uptake on imaging in order to minimize side-effects in irradiated tissue. The unique overexpression on prostate cancer cells makes PSMA the most attractive target for the delivery of imaging agents [10]. Several clinical studies with PSMA as the target have been reported with encouraging results [71,72]. Bombesin-like radiopharmaceuticals, which are natural ligands of GRPR, can be relatively easy synthesized in large quantities and have shown promising results in several clinical studies [70]. However, due to its background staining, more careful selection of the protocol may be required for optimal targeting. Only a few studies consider VEGF and EpCAM-based diagnostics for targeted cancer imaging [73–75], but based on the results reported in this study, both antigens can be used for the detection of locally recurrent prostate cancer.

## 3. Experimental Section

### 3.1. Materials

Patients in our retrospective study were diagnosed with locally recurrent prostate cancer based on PSA relapse and transrectal ultrasound-guided prostate biopsies. Prostate cancer specimens were collected of 17 patients who underwent salvage prostatectomy (The Netherlands Cancer Institute, Antoni van Leeuwenhoek Hospital Amsterdam (NKI/AVL)), because of locally recurrent prostate cancer after brachytherapy (4 patients) or external beam radiotherapy (13 patients). All tissue specimens were anonymous and encoded with a unique code. According to Dutch law, no further Institutional Review Board approval was required (http://www.federa.org). Pretreatment biopsies were not available.

### 3.2. Immunohistochemistry

Formalin-fixed, paraffin-embedded blocks of prostate tissue were cut into 4-mm-thick sections and mounted on Starfrost microscope slides. Hematoxylin and eosin stained sections were graded by an experienced pathologist (Stefano Rosati), based on the criteria of the Gleason grading system. Remaining sections were processed for immunohistochemistry.

#### 3.2.1. PSMA

After deparaffinization, antigen retrieval was performed by heating microwave (700 W) for 20 min in a 10 mM citrate buffer at pH 6.0, with a cool down period of 20 min afterwards. Endogenous peroxidase was blocked with 0.3% hydrogen peroxide in phosphate-buffered saline (PBS) for 20 min. Slides were than incubated with the primary anti-human-PSMA mouse monoclonal antibody, YPSMA-1 (Abcam, Cambridge, UK), diluted at 1:400 in 1% bovine serum albumin/phosphate-buffered saline (1% BSA/PBS) for 1 h at room temperature. The secondary step consisted of incubation with rabbit anti-mouse antibody conjugated to polymer-horseradish peroxidase (DAKO, Glostrup, Denmark), diluted at 1:100 in 1% BSA/PBS with 1% AB serum. For the tertiary step, goat anti-rabbit antibody conjugated to polymer-horseradish peroxidase (DAKO, Glostrup, Denmark) was used, diluted at 1:100 in 1% BSA/PBS with 1% AB serum. Both the secondary and tertiary step required incubation for 30 min at room temperature. Next, the slides were immersed for 10 min in a solution of 0.05% 3,3′-diaminobenzidine (Sigma-Aldrich, St. Louis, MO, USA) and 0.03% hydrogen peroxide in PBS for the visualization of the signal as brown staining. After washing with demineralized water, the slides were slightly counterstained with hematoxylin, dehydrated and mounted with Eukitt mounting medium (Sigma-Aldrich, Steinheim, Germany).

#### 3.2.2. GRPR

After deparaffinization, antigen retrieval was performed by heating microwave (700 W) for 20 min in 0.1 M Tris/HCl buffer at pH 9.0, with a cool down period of 20 min afterwards. Endogenous peroxidase was blocked with 0.3% hydrogen peroxide in Tris-buffered saline (TBS) for 20 min. Slides were then incubated with a normal goat serum diluted at 1:10 in TBS for 30 min at room temperature. Afterwards, the slides were incubated with the primary anti-human-GRPR rabbit polyclonal antibody, ab39963 (Abcam, Cambridge, UK), diluted at 1:250 in 1% BSA/TBS overnight at 4 °C. Only a secondary step with goat anti-rabbit antibody conjugated to polymer-horseradish peroxidase (DAKO, Glostrup, Denmark) was applied, diluted at 1:100 in 1% BSA/TBS with 1% AB serum for 60 min at room temperature. Next, the slides were immersed for 10 min in a solution of 0.05% 3,3′-diaminobenzidine (Sigma-Aldrich, Steinheim, Germany) and 0.03% hydrogen peroxide in PBS for the visualization of the signal as brown staining. After washing with demineralized water, the slides were slightly counterstained with hematoxylin, dehydrated and mounted with Eukitt mounting medium (Sigma-Aldrich, Steinheim, Germany).

#### 3.2.3. EpCAM

After deparaffinization, antigen retrieval was performed by incubation with 0.1% protease for 30 min at room temperature. Endogenous peroxidase was blocked with 0.3% hydrogen peroxide in PBS for 20 min. Slides were than incubated with the primary mouse monoclonal anti-EpCAM antibody (Clone VU-1D9, Leica Biosystems, Newcastle, UK) diluted at 1:100 in 1% BSA/PBS for 1 h at room temperature. The secondary step consisted of incubation with rabbit anti-mouse antibody conjugated to polymer-horseradish peroxidase (DAKO, Glostrup, Denmark), diluted at 1:100 in 1% BSA/PBS with 1% AB serum. For the tertiary step, goat anti-rabbit antibody conjugated to polymer-horseradish peroxidase (DAKO, Glostrup, Denmark) was used, diluted at 1:100 in 1% BSA/PBS with 1% AB serum. Both the secondary and tertiary step required incubation for 30 min at room temperature. Next, the slides were immersed for 10 min in a solution of 0.05% 3,3′-diaminobenzidine (Sigma-Aldrich, Steinheim, Germany) and 0.03% hydrogen peroxide in PBS for visualization of the signal as brown staining. After washing with demineralized water, the slides were slightly counterstained with hematoxylin, dehydrated and mounted with Eukitt mounting medium (Sigma-Aldrich, Steinheim, Germany).

#### 3.2.4. VEGF

After deparaffinization, microwave antigen retrieval (700 W) was performed for 20 min in 10 mM Tris/1 mM EDTA buffer at pH 9.0, with a cool down period of 20 min afterwards. Endogenous peroxidase was blocked with 0.3% hydrogen peroxide in PBS for 20 min. Slides were incubated with a normal goat serum diluted at 1:10 in PBS for 30 min at room temperature. The primary step consisted of incubation with rabbit anti-human antibody VEGF A-20 sc-152, (Santa Cruz Biotechnology, Santa Cruz, CA, USA) diluted at 1:200 in 1% BSA/PBS for 1 h at room temperature. Only a secondary step with goat anti-rabbit antibody conjugated to polymer-horseradish peroxidase (DAKO, Glostrup, Denmark) was applied, diluted at 1:100 in 1% BSA/TBS with 1% AB serum for 30 min at room temperature. Next, the slides were immersed for 10 min in a solution of 0.05% 3,3′-diaminobenzidine (Sigma-Aldrich, Steinheim, Germany) and 0.03% hydrogen peroxide in PBS for the visualization of the signal as brown staining. After washing with demineralized water, the slides were slightly counterstained with hematoxylin, dehydrated and mounted with Eukitt mounting medium (Sigma-Aldrich, Steinheim, Germany).

### 3.3. Assessment of Staining Patterns

The assessment of staining patterns was performed as described before [8]. For each antigen, a pathologist (Stefano Rosati) blinded to clinical and pathological data, scored the staining intensity (0 = no staining; 1+ = weak staining; 2+ = moderate staining; 3+ = strong staining) of tumor areas for all the specimens. Specimens in which one or more tumor areas with different staining intensities were present were scored for the most prevalent intensity. Specimens with focal uptake amidst negative cancer tissue scored 1+. Furthermore, different patterns of immunoreactivity were observed and documented. To evaluate background staining, all specimens were evaluated in a field that contained both prostate cancer and stroma. Tumor distinctiveness was assessed for each antigen by subtracting the staining intensity of stroma from the staining intensity of prostate cancer.

## 4. Conclusions

The current study is the first to present data on the expression of PSMA, EpCAM, VEGF and GRPR in locally recurrent prostate cancer after brachytherapy or external beam radiotherapy. Based on the absence of stromal staining, PSMA, EpCAM and VEGF show high tumor distinctiveness. GRPR has a very low tumor distinctiveness. Therefore, PSMA, EpCAM and VEGF can be used as targets for the bioimaging of recurrent prostate cancer after EBRT to exclude metastatic disease and/or to plan local salvage therapy.

## Figures and Tables

**Table 1.** Gleason sum scores, therapy characteristics and staining intensities of the antibodies in the salvage prostatectomy specimens.

Patient No.	Radiotherapy (Dose in Gy)	Hormonal Status Prior to Salvage Prostatectomy	Interval between Radiotherapy and Salvage Prostatectomy (Months)	Stage	Gleason	PSMA	EpCAM	VEGF	GRPR
1	EBRT (Dose unknown)	LHRH + AA	51	pT3b	7	++	+	++	+++
2	Brachytherapy (HDR)	None	45	pT2c	8	+	++	++	+++
3	EBRT (70)	LHRH + AA	58	pT3b	8	+	−	+	+++
4	EBRT (70)	None	24	pT3a	7	+	+	−	++
5	EBRT (Dose unknown)	None	80	pT2c	7	++	+	−	+
6	Brachytherapy (LDR)	None	47	pT2c	cnd	+	+	−	+++
7	EBRT (70)	LHRH + AA	120	pT3a	7	+++	−	+	+
8	EBRT (66)	None	31	pT3b	8	+	−	+	+
9	EBRT (66)	None	78	pT4	7	+++	+++	+	+++
10	EBRT (66)	None	48	pT3b	8	+++	++	++	+++
11	EBRT (Dose unknown)	Unknown	63	pT3b	7	+++	+++	+++	++
12	Brachytherapy (LDR)	None	41	pT4	7	+++	+++	+	++
13	EBRT (70)	None	49	pT3a	8	+	++	++	++
14	EBRT (68)	LHRH	58	pT3a	6	+++	+++	++	+++
15	Brachytherapy (LDR)	AA	88	pT3b	8	+++	++	++	+++
16	EBRT (68)	None	13	pT3b	6	+	++	+	++
17	EBRT (70)	LHRH	34	pT3b	10	+++	++	+	++

cnd, could not be determined; EBRT, external beam radiotherapy; HDR, high dose rate; LDR, low dose rate; LHRH, luteinizing-hormone-releasing hormone agonist; AA, androgen receptor antagonist.

**Table 2.** Immunohistochemical staining intensity of prostate cancer and stroma.

Staining Intensity	PSMA Prostate Cancer	PSMA Stroma	EpCAM Prostate Cancer	EpCAM Stroma	VEGF Prostate Cancer	VEGF Stroma	GRPR Prostate Cancer	GRPR Stroma
0	0 (0%)	17 (100%)	3 (17.7%)	17 (100%)	3 (17.7%)	17 (100%)	0 (0%)	0 (0%)
1+	7 (41.2%)	-	4 (23.5%)	-	7 (41.2%)	-	3 (17.7%)	3 (17.7%)
2+	2 (11.8%)	-	6 (35.3%)	-	6 (35.3%)	-	6 (35.3%)	8 (47.0%)
3+	8 (47.0%)	-	4 (23.5%)	-	1 (5.8%)	-	8 (47.0%)	6 (35.3%)
Overall+	17/17 (100%)	0/17 (0%)	14/17 (82.3%)	0/17 (0%)	14/17 (82.3%)	0/17 (0%)	17/17 (100%)	17/17 (100%)

**Table 3.** Tumor distinctiveness.

Tumor distinctiveness	PSMA	EpCAM	VEGF	GRPR
0	-	3 (17.7%)	3 (17.7%)	15 (88.2%)
1	7 (41.2%)	4 (23.5%)	7 (41.2%)	2 (11.8%)
2	2 (11.8%)	6 (35.3%)	6 (35.3%)	-
3	8 (47.0%)	4 (23.5%)	1 (5.8%)	-

Tumor distinctiveness = staining intensity tumor – staining intensity stroma.
